# The Edmonton Frail Scale as a screening score for frailty in oncogeriatrics

**DOI:** 10.3389/fmed.2024.1466366

**Published:** 2024-09-30

**Authors:** Julia De Schrevel, Clémentine Faucon, François-Xavier Sibille, Laura Dumont, Francois R. Herrmann, Héloïse Rouvière, Sandra De Breucker

**Affiliations:** ^1^Geriatric Department, Hôpital Universitaire de Bruxelles, Brussels, Belgium; ^2^Geriatric Department, CHU UCL, Namur, Belgium; ^3^Institute of Health and Society (IRSS), Brussels, Belgium; ^4^Oncologic Department, Hôpital Universitaire de Bruxelles, Brussels, Belgium; ^5^Department of Rehabilitation and Geriatric, Geneva University Hospitals and University of Geneva, Geneva, Switzerland

**Keywords:** comprehensive geriatric assessment, Edmonton Frail Scale, cancer, oncogeriatrics, frailty

## Abstract

**Introduction:**

The comprehensive geriatric assessment (CGA) is recommended for assessing frailty in older cancer patients but is time-consuming. The G8 screening tool was developed to select frail patients requiring CGA to optimize resources. The Edmonton Frail Scale (EFS) is another frailty scale validated for preoperative frailty screening, but scarcely studied in the field of oncogeriatrics. In this study, we examined the added value of the EFS in older cancer patients already considered as frail by the G8, by analyzing the association of EFS with CGA adjusted for age, gender, metastatic stage and comorbidity. We also analyzed the association of EFS with the one-year mortality rate after adjusting for cancer type and metastatic stage.

**Methodology:**

This retrospective study included patients aged over 70 years old with a new diagnosis of cancer, considered as potentially frail according to the G8 and who had had a CGA (*N* = 380).

**Results:**

The EFS identified 329 (86.58%) patients as frail and having a statistically significant predicted number of pathological components on the CGA (*r* = 0.64, *p* < 0.001). When adjusted for age, sex, comorbidity, and metastatic stage, the EFS was independently associated with the CGA (*p* < 0001), as well as with comorbidity (*p* = 0.004). The patients who died in the first year (43%) had a significantly higher mean EFS score (8/17) than living patients (6/17) (*p* < 0.0001). After adjustment for cancer type and stage, EFS independently predicted one-year mortality (OR 1.17; 95% CI 1.08–1.28; pseudo *R*^2^ = 0.228, *p* < 0.001).

**Discussion:**

The EFS is a reliable tool for predicting frailty identified by the CGA in an older cancer population pre-selected as frail by the G8. EFS is an independent predictor of one-year mortality after adjustment for confounding factors. Validation of the EFS as a screening tool for frailty in cancer requires further studies to assess its performance in patients with normal G8 scores.

## 1 Introduction

Due to an ageing population, increased prevalence of cardiovascular risk factors, and environmental pollution, around 35 million new cases of cancer are expected by 2050 worldwide, representing a 77% increase from the 20 million cases estimated in 2022 (1). Among them, around two-thirds are expected to occur in people over the age of 65 (2).

The health status of older people is heterogeneous, which can be partly explained by the concept of frailty. Frailty is a state of vulnerability to stress, linked to the depletion of the homeostatic reserves (3). The performance status, which is usually used in oncology to characterise a patient’s state of health appears to be insufficient to define an older person’s state of health, as it only captures the global functional status, without addressing the broader range of geriatric frailties that can significantly affect a patient’s ability to tolerate and achieve the oncological treatment (4). Additionally, frail older patients are often under-represented in oncology clinical trials, contributing to the lack of clear therapeutic guidelines for these patients (5, 6).

Scientific bodies in oncology recommend a comprehensive geriatric assessment (CGA) to evaluate the health status of older cancer patients (7, 8). This assessment has been shown to be effective in predicting functional decline (9), survival (10, 11) and treatment-related toxicities (12) and can help to manage non oncological comorbidities and refine the cancer treatment plan (13). However, a CGA is time-consuming and, for this reason, is not often performed in practice due to limited resources (14, 15). To meet this challenge and identify frail patients who would most benefit from a CGA, the geriatric 8 (G8) screening tool was proposed (16). The G8 consists of 8 questions assessing nutrition, cognitive and mood functions, age, polymedication, and self-perception of health. The G8 offers the advantage to be brief and easy to administer in a clinical setting, even by non-geriatric healthcare professionals, and it has a good sensitivity. However, despite these benefits, its implementation in clinical practice is challenging. The most cited barriers to using the G8 include oncologists’ subjective perception that the G8 does not add value to the usual diagnostic workup, a lack of awareness among some oncologists about geriatric frailty or their failure to collaborate with geriatricians, as well as limited time and coordination to determine who will administer the G8 in the care pathway (17, 18).

The Edmonton Frail Scale (EFS) is a frailty screening scale initially developed to predict complications of cardiac surgery in older people. It was validated in a study with 158 older people, either in or outpatient geriatric clinics (19). This scale provides a score on 17 points and stratifies patients into four categories (not frail, mild frailty, moderate frailty, and severe frailty). With questions covering cognitive, functional, social, nutritional, mood, and continence status, as well as medication and a general health assessment, the EFS provides more multifaceted information than the G8 for geriatric screening and is quick to administer, taking around 5 min. To date, the number of studies evaluating the value of the EFS as a screening tool for older cancer patients is limited (20–26).

Given the lack of studies validating the EFS as a screening tool for frailty in oncogeriatrics, we aimed to assess the performance of the EFS by comparing it to the gold standard, the CGA. This assessment was carried out in patients aged 70 and over, newly diagnosed with a solid cancer or hematological malignancy, who had an impaired G8 score and for whom treatment was being considered.

In addition, we examined the association between frailty as determined by the EFS and 1-year all-cause mortality, adjusting for cancer type and metastatic stage.

## 2 Materials and methods

### 2.1 Study design

This retrospective study was conducted in two academic centers in Brussels and included 380 patients already classified as potentially frail by the G8 score administered by the oncological teams and were referred to the geriatric teams for a comprehensive geriatric assessment between March 1st, 2018 and April 30th, 2023. The study was approved by the local ethics committee of each participating centre (Erasme Hospital and Jules Bordet Institute) in February 2024 under the final reference number SRB2023274. The Erasme Hospital acted as the central ethics committee.

### 2.2 Patient identification

The inclusion criteria were as follows: patients had to be aged 70 years or older and have a confirmed new diagnosis of a solid cancer or hematological malignancy, confirmed by histological or radiological evidence. The G8 score had to be of 14/17 or lower. Additionally, participants had to be eligible for the proposed curative or palliative treatment, such as surgery, radiotherapy, chemotherapy, hormone therapy, targeted therapy, radiotherapy, endoscopic and/or local treatment.

The exclusion criteria were the presence of two or more neoplasia, namely another active cancer or a history of another cancer, or the patient’s refusal to use his/her medical data for research purposes, as documented in the medical records.

### 2.3 Data collected

#### 2.3.1 Patient characteristics

Age, gender, place of residence (nursing home or home), number of medications taken at home, body mass index (BMI) and the Edmonton Frail Scale score were collected.

#### 2.3.2 Oncological characteristics

Cancer types were classified as follows: gastro-intestinal; pancreatic, liver, and biliary tract; lung and mesothelioma; breast; gynecological (excluding breast); prostate; urological (excluding prostate); hematological malignancies; otolaryngological and oral neoplasia; other cancers and cancers of unknown origin. The proposed therapy and the indication for curative or palliative treatment were also documented. Cancers were also classified as either “advanced disease” in the presence of metastases for solid cancers or a poor prognostic score for hematologic malignancies, or as “localised disease” if not.

#### 2.3.3 Comprehensive geriatric assessment

The CGA included assessment of domains referenced in the ONCODAGE study by Dale et al. (14). These domains included Activities of Daily Living (ADL) assessed by the Katz score, Instrumental Activities of Daily Living (IADL) using the Lawton score, the Geriatric Depression Scale (GDS) with 4 or 15 items, the Mini-Mental State Examination (MMSE) or Montreal Cognitive Assessment (MoCA), the Mini Nutritional Assessment (MNA) or MNA-short form (MNA-sf), the level of comorbidity assessed by the Cumulative Illness Rating Scale-Geriatrics (CIRS-G), and the Timed Up and Go (TUG), or Short Physical Performance Battery (SPPB) (27). The choice of the tool used in each domain was left to the discretion of the examiner.

A domain was considered pathological if the score obtained was below the threshold defined in the literature (Table 1). MoCA scores were converted to theoretical MMSE scores in accordance with the reference article by Fasnacht et al. (28). In cases where a patient’s condition did not allow a mobility or cognitive test to be performed, the score was considered as pathological. The sum of the pathological domains was calculated, and the CGA was considered pathological if there was at least one abnormal score (27).

**Table 1:** Comprehensive geriatric assessment.

Domains	Scale	Thresholds
Functional	ADL	≤5/24
	IADL	≤7/8
Comorbidities	CIRS-G	0–56
Cognitive	MMSE	≤23/30
	MOCA	≤18/30
Nutritional	MNA	≤23.5/30
	MNA-sf	≤11/14
Mobility	Timed up and Go	>20 s
	SPPB	≤8/12
Mood	GDS 15	≥6/15
	GDS 4	≥1/4

ADL, Activities of Daily Living; IADL, Instrumental Activities of Daily Living; CIRS-G, Cumulative Illness Rating Scale for Geriatrics; MMSE, Mini Mental Scale Examination; MOCA, Montreal Cognitive Assessment; MNA, Mini Nutritional Assessment; SPPB, Short Physical Performance Battery; GDS, Geriatric Depression Scale.

We also documented additional parameters, including the Eastern Cooperative Oncology Group (ECOG) performance status, visual analogue fatigue score, the number of patients with at least one fall in the last 6 months and the grip strength measured using a Jamar dynamometer: muscle strength was considered low if below 16 kg for women and 27 kg for men (29). Furthermore, we assessed the 4-year prognosis using the Lee score (30) and predicted the risk of severe chemotoxicity (grade 3 or higher) using the CARG score for patients with a chemotherapy treatment plan (12).

#### 2.3.4 Follow up

The follow up data included overall mortality at 1 year.

### 2.4 Statistical analysis

The statistical analyses were carried out using STATA^®^ version 16, College Station, Texas.

The sample was described by clinical and demographic characteristics for total group, frail and non-frail patient groups among the CGA. Continuous variables were presented as means ± standard deviations in case of parametric distribution of variables, or as medians with interquartile ranges (25th percentile to 75th percentile) in case of non-parametric distribution of variables. Categorical and discrete variables were reported as absolute values and percentages. Frail group and non-frail group were compared for clinical and oncological characteristics.

The diagnostic performance of the EFS for frailty screening was assessed by comparing pathological EFS with pathological CGA and expressed in EFS sensitivity, specificity, positive predictive value, negative predictive value, and negative post-test probability. A ROC curve was then drawn.

We also analysed the correlation of EFS and CGA, and of EFS and G8 using Pearson’s correlation, and we made a linear regression analysis of CGA with EFS in univariable and multiple regression adjusted for sex, age, the comorbidity assessed by CIRS-G, and the presence of metastases. The coefficient of determination (adjusted *r*^2^) adjusted for the number of variables present in the model is provided to assess the proportion of the variance explained by the model.

The prognostic value of the EFS was assessed by evaluating the overall one-year mortality: we compared mean EFS scores between deceased and living patients using a student’s *t*-test and then analysed the association between EFS and one-year mortality using univariate logistic regression. We then performed multiple regression analysis adjusting for cancer type and stage (local, advanced, or unknown). All assumptions of the multivariable analysis were verified. Each EFS frailty category was also examined individually in terms of prediction of one-year mortality using Fisher’s chi-square test. Finally, we performed an analysis to determine the sensitivity and specificity of each EFS point using the Youden index to identify a threshold score predictive of one-year mortality. A *p*-value less than 0.05 was considered as significant in all analyses.

## 3 Results

### 3.1 Recruitment

**Figure 1:**
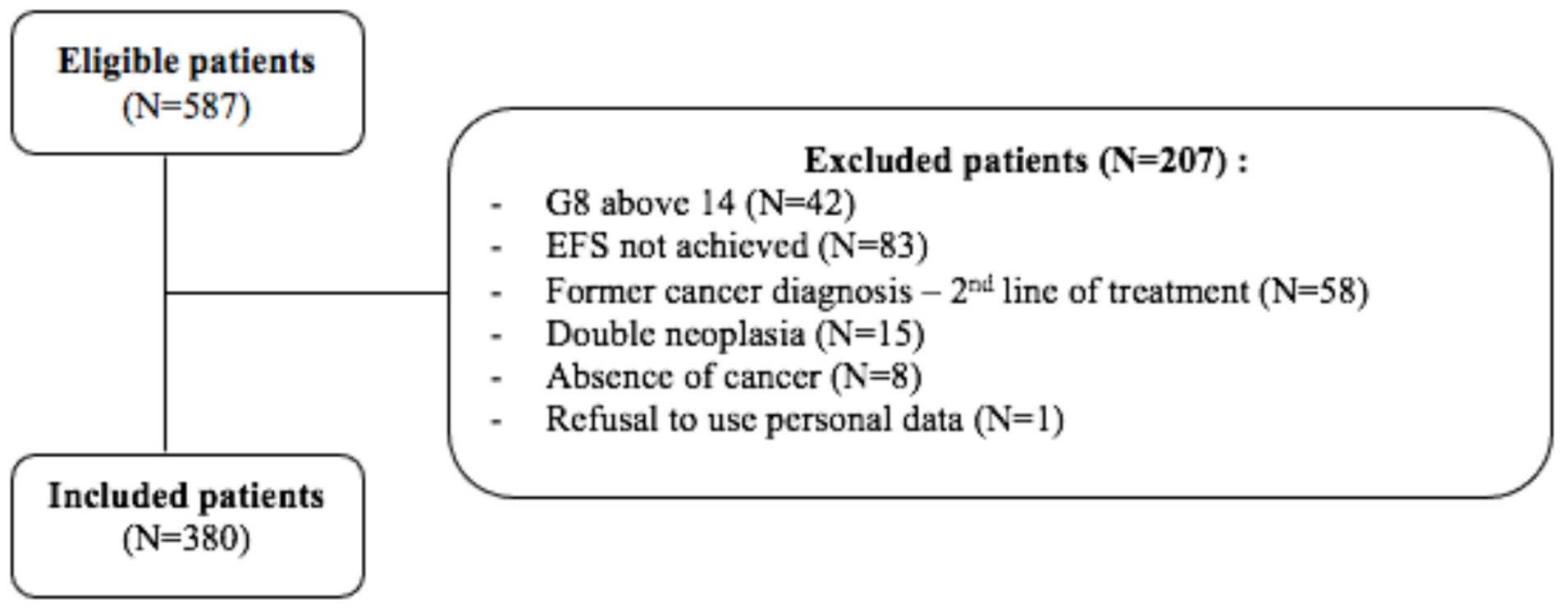
Flow chart for patient inclusion and exclusion.

During the study period, a total of 587 patients were evaluated. Of these, 380 patients were included while 207 were excluded for the reasons described in Figure 1.

### 3.2 Descriptive data on the study population

Descriptive, oncological and CGA data are presented in Tables 2–4.

**Table 2:** Descriptive data on the study population.

	*N*	Total group (*n* = 380)	Frail CGA group (*n* = 355)	Non frail CGA group (*n* = 23)	*p*-value
Age (years)	380	82 [77–85]	82 [77–85]	82 [75–84]	0.371
Female gender	380	177 (46.6)	185 (52.1)	18 (78.3)	0.015
Living at home	380	343 (90.3)	318 (89.6)	23 (100)	0.265
Number of daily medications	380	7 ± 3	7 ± 4	5 ± 3	0.060
BMI (kg/m^2^)	380	24.8 [21.7–28.1]	24.8 [21.7–28.0]	26.9 [23.8–29.4]	0.046
ECOG	380				<0.001
0		72 (18.9)	56 (15.8)	16 (69.6)	
1		119 (31.3)	113 (31.8)	6 (26.1)	
2		83 (21.8)	82 (23.1)	1 (4.3)	
3		92 (24.3)	90 (25.4)	0	
4		14 (3.7)	14 (3.9)	0	
G8 (/17)	380	9.5 [7.5–12]	9.5 [7.5–11.5]	13 [10.5–14]	<0.001
EFS (/17)	380	7 ± 3	7 ± 3	3 ± 2	<0.001
0–3 (not frail)		51 (13.4)	38 (10.7)	13 (56.5)	<0.001
4–5 (mild frailty)		66 (17.4)	58 (16.3)	8 (34.8)	0.024
6–8 (moderate frailty)		128 (33.7)	125 (35.2)	2 (8.7)	0.009
≥9 (severe frailty)		135 (35.5)	134 (37.8)	0 (0)	<0.001
Fatigue (/10)	275	4.9 ± 2.6	5.0 ± 2.6	2.6 ± 2.3	<0.001
At least one fall <6 months	355	134 (37.8)	131 (39.6)	3 (13.6)	0.015
Probable sarcopenia	288	144 (50.0)	137 (51.1)	5 (27.8)	0.055
CIRS G (/56)	380	15 [12–19]	16 [12–19]	14 [11–15]	0.018
Lee score (/27)	368	12 ± 3	12 ± 3	9 ± 3	<0.001

Data are expressed in mean (± standard deviation) or median [p25–p75] or *n* (%). BMI, body mass index; ECOG, Eastern Cooperative Oncology Group; EFS, Edmonton Frail Scale; CIRS G, Cumulative Illness Rating Scale for Geriatrics.

**Table 3:** Oncological data.

		Total group (*n* = 380)	Frail CGA group (*n* = 355)	Non frail CGA group (*n* = 23)	*p*-value
	*N*	*n* (%)	*n* (%)	*n* (%)	
Type of cancer	380				0.434
Gastro-intestinal (other than pancreas, liver, and biliary tract)		66 (17.4)	60 (16.9)	6 (26.1)	
Pancreas, liver, and biliary tract		63 (16.6)	61 (17.2)	2 (8.7)	
Breast		47 (12.4)	43 (12.1)	3 (13.0)	
Urological (other than prostate)		43 (11.3)	41 (11.6)	1 (4.4)	
Lung and mesothelioma		42 (11.0)	39 (11.0)	3 (13.0)	
Prostate		42 (11.0)	37 (10.4)	5 (21.7)	
Hematological malignancies		34 (9.0)	33 (9.3)	1 (4.4)	
Otolaryngological and oral neoplasia		15 (4.0)	15 (4.2)	0 (0)	
Other cancers		13 (3.4)	11 (3.1)	2 (8.7)	
Gynaecological (other than breast)		11 (2.9)	11 (3.1)	0 (0)	
Primitive of unknown origin		4 (1.0)	4 (1.1)	0 (0)	
Stage	378				0.125
Localised disease		139 (36.6)	126 (35.5)	13 (56.5)	
Advanced disease		155 (40.8)	147 (41.4)	6 (26.1)	
Unknown stage		86 (22.6)	82 (23.1)	4 (17.4)	
Therapeutic plan	378				
Curative		136 (36.0)	119 (33.5)	17 (74.0)	<0.001
Palliative		72 (19.0)	69 (19.4)	3 (13.0)	0.449
Unknown/not applicable (no treatment proposed)		170 (45.0)	167 (47.1)	3 (13.0)	0.032
Interruption of treatment at 1 year	127	31 (24.4)	29 (25.2)	2 (18.2)	0.605

**Table 4:** Pathological domains of the comprehensive geriatric assessment.

	*N*	Mean or median values	Pathological domain *n* (%)
Functional			
ADL (/24)	379	7 [6–9]	236 (62.2)
IADL (/8)	254	5 ± 3	188 (74.0)
Cognitive	338		110 (32.5)
MMSE (/30)	238	26 [21–28]	
MOCA (/30)	100	23 [17–25]	
Nutritional	362		212 (58.6)
MNA (/30)	291	19 [15–23]	
MNA sf (/14)	71	7 [6–9]	
Mobility	320		111 (34.7)
Timed Up and Go (seconds)	284	15 [11–21]	
SPPB (/12)	166	6 ± 3	
Mood	343		122 (35.5)
GDS 4	51	1 ± 1	
GDS 15	292	4 [2–6]	
Pathological geriatric domains (/6)	380	3 ± 1	357 (93.9)

Data are expressed in mean (± standard deviation) or median [p25–p75] or *n* (%). ADL, Activities of Daily Living; IADL, Activities of Daily Living; MMSE, Mini Mental Scale Examination; MOCA, Montreal Cognitive Assessment; MNA, Mini Nutritional Assessment; SPPB, Short Physical Performance Battery; GDS, Geriatric Depression Scale.

The prevalence of frailty as assessed by CGA was 94%, with a median number of geriatric syndromes of 3 [IQR 2–3], and the prevalence of frailty by EFS was 87%, with a mean score of 7 ± 3 points.

Ninety-four percent (*n* = 357) of potentially frail patients, according to G8, had an abnormal CGA.

Among the 6% (*n* = 23) who had a normal CGA, 13 patients (56.5%) were considered not frail by EFS, 8 (34.8%) had mild frailty, 2 (8.7%) had moderate frailty and none had severe frailty.

**Figure 2:**
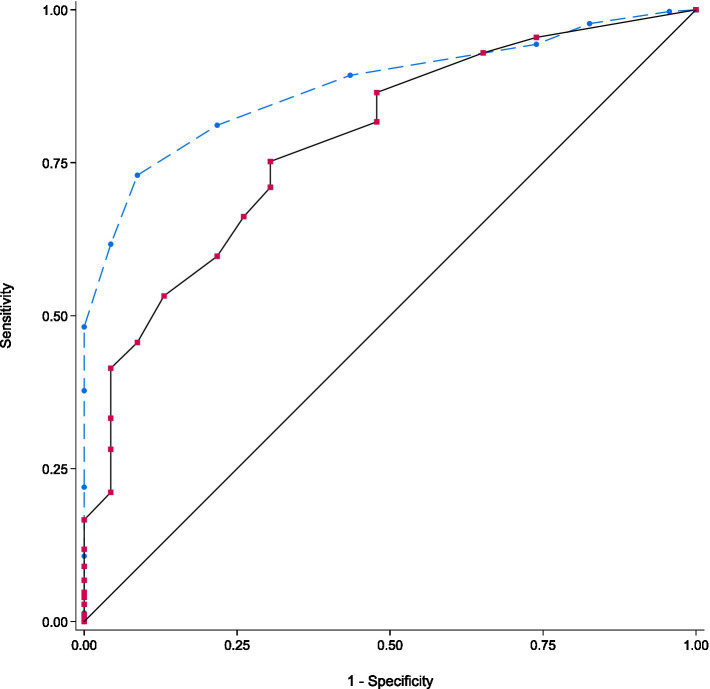
ROC curve comparing Edmonton Frail Scale with the gold standard (CGA).

Forty-one percent (*n* = 155) of patients had advanced disease and 36% (*n* = 139) had localised disease. More patients had a curative treatment plan (*n* = 136; 36%) rather than a palliative treatment plan (*n* = 72; 19%), the remaining patients being considered for therapeutic abstention or for an unknown treatment plan. The EFS sensitivity was 89.3% and specificity 43.5%. The positive predictive value was 96%, the negative predictive value was 20.8% and the negative post-test probability was 79.2%. The area under the curve was 0.73, indicating a fair performance of EFS to predict frailty (Figure 2).

### 3.3 Correlation and regression analyses between EFS and CGA

**Figure 3:**
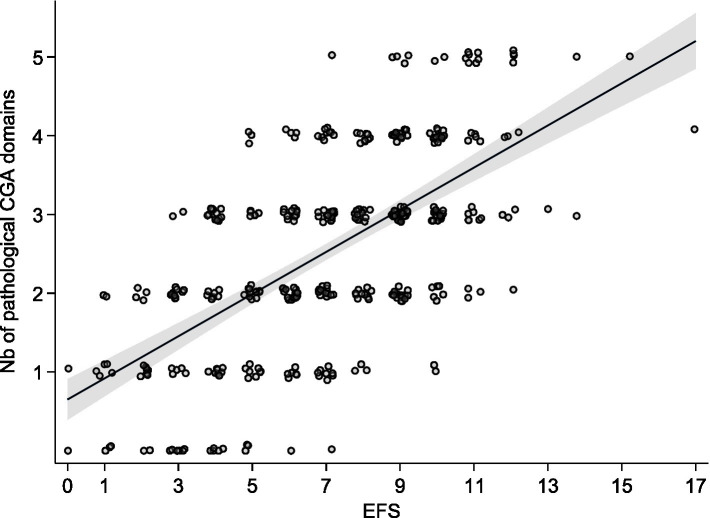
Pearson’s correlation between the EFS and the number of pathological domains in the CGA.

**Figure 4:**
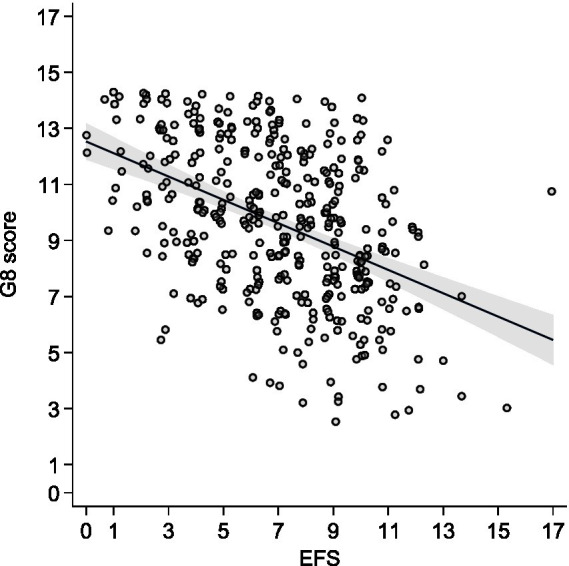
Pearson’s correlation between the EFS and the G8 score.

The Edmonton Frail Scale was significantly and positively correlated with the CGA (*r* = 0.6403, *p* < 0.0001) and significantly and negatively correlated with the G8 (*r* = −0.277, *p* < 0.0001) (Figures 3, 4).

In univariate analysis, the EFS was significantly associated with the CGA [*β* coeff = 0.27; 95% CI (0.23–0.30); *p* < 0.0001], with an adjusted *r*^2^ = 0.392. When adjusted for age, sex, comorbidity, and metastatic stage, the EFS was independently associated with the CGA [Adj *β* coeff = 0.26 (0.22–0.29); *p* < 0001], as well as with comorbidity [Adj *β* coeff = 0.027 (0.008–0.047); *p* = 0.004], with an adjusted *r*^2^ = 0.402.

### 3.4 Prognostic value of the EFS

Forty-three percent of the patients had died within 1 year (*n* = 165). Deceased patients had a significantly higher EFS than living patients (7.8 ± 2.6 and 6.4 ± 3 respectively, *p* < 0.0001).

In univariate analysis, we found a significant association between the EFS and one-year mortality (OR 1.20, 95% CI 1.12–1.30, *p* < 0.0001). This association remained significant in multiple regression analysis after adjustment for cancer type and stage (OR 1.17, 95% CI 1.08–1.28, *r*^2^ = 0.228, *p* < 0.001).

Compared to other types of cancer, mortality was multiplied by 3.19 for pancreatic-liver-biliary cancers and by 1.81 for lung and related cancers. Conversely, the risk of death was reduced by 74% for breast cancer and by 73% for prostate cancer. Compared with localised disease, the risk of death was multiplied by 4.26 in the presence of advanced disease. In addition, patients with an unknown disease stage had a mortality rate 1.53 times higher than those with localised disease (Table 5).

**Table 5:** Multivariable logistic regression analysis of factors associated with 1-year mortality.

	Odds ratio	95% CI	*p*-value
Edmonton Frail Scale	1.17	1.08–1.28	<0.001
Cancer types			
Pancreas, liver and biliary tract	3.19	1.69–6.20	<0.001
Lung and mesothelioma	1.81	0.86–3.86	0.120
Breast	0.26	0.10–0.60	<0.001
Prostate	0.27	0.10–0.60	<0.001
Stage			
Localised disease	1	1	
Advanced disease	4.26	2.48–7.43	<0.001
Unknown stage	1.53	0.82–2.86	0.183

Analysing each EFS category separately, we observed a significant difference in one-year mortality between robust patients and the rest of the sample (21.5% vs. 46.8%, *p* = 0.001), as well as between mild frailty patients (31.8% vs. 45.9%; *p* = 0.036), or severe frailty patients (56.3% vs. 36.3%, *p* < 0.0001) and the rest of the sample. There was no significant difference between moderately frail subjects (44.5% vs. 42.8%, *p* = 0.756) and the rest of the sample.

The EFS threshold predictive of one-year mortality was 7 or more, with a sensitivity of 72% and a specificity of 52%, as determined by the Youden index.

## 4 Discussion

We showed that all but 23 (6%) of potentially frail old cancer patients according to G8 had an abnormal CGA, among which 13 (56%) were considered not frail by EFS; and that EFS was independently associated with CGA, comorbidity and 1-year mortality.

In our cohort, the prevalence of frailty defined by the EFS was higher (87%) than typically cited in the literature, which ranges from 34 to 60% (21–23). This might be explained by the preselection of patients already considered frail by the G8. Another possible explanation for this difference is that some studies used different thresholds for the EFS: ≥6 (21), ≥7 (22, 23, 26), or ≥8/17 (20). We used the threshold of ≥4 and thus the prevalence of frailty may have been overestimated.

In comparison with a CGA, the gold standard, we found a sensitivity of the EFS of 89% and a specificity of 43.5%. Most of the patients had an abnormal CGA (94%), and while 23 patients (6%) had a normal CGA, most of the latter (91%) being classified as not frail or mildly frail by the EFS.

Since our patients were pre-selected as potentially frail due to a pathological G8 score and considering that the average sensitivity and specificity of the G8 was estimated at 76.5 and 64.4% respectively, in the ONCODAGE study, we can presume that the EFS adds 12.5% of sensitivity to detecting frailty, whereas it loses 20.9% of specificity (16). This increase in sensitivity is important in older people with cancer, as it allows a more accurate exclusion of the presence of a frailty syndrome, which is predictive of overall, cancer-related and disease-free survival, treatment-related complications, premature treatment discontinuation and healthcare resource utilisation for both solid tumours and hematologic malignancies (31–35). In addition, the positive predictive value of 96% indicates that the EFS provides even greater reliability as to the presence of frailty when the G8 test is positive. From a clinical point of view, our results suggest that even in already frail patients with cancer, EFS could help avoid unnecessary geriatric assessments, thus better allocating geriatric evaluation resources to patients with confirmed frailty, the EFS being quicker to administer and informative on geriatric domains that are not evaluated by the G8, such as cognitive function, physical performance, continence, and social support.

We observed a significant correlation between the EFS and the CGA. This was already shown in a previous study involving 300 older cancer patients treated with radiotherapy. Using an EFS threshold of ≥6, the sensitivity of the EFS was 97%, and the specificity was 57% (21). However, the gold standard used corresponded to the presence of at least two pathological domains, and three additional domains were added compared to the ONCODAGE study, namely comorbidities according to the modified Charlson score, polypharmacy, and the number of falls that occurred in the last 6 months.

In our study, we did not include comorbidities as a domain of the CGA, since all patients had at least one comorbidity with a CIRS-G score ≥3 due to the presence of the cancer diagnosis, as it did not seem methodologically appropriate to include the CIRS-G score, and consider cancer as a comorbidity. This methodological approach differs from the ONCODAGE study and can lead to an underestimation of frailty as assessed by the CGA. This point highlights the lack of consensus regarding the identification of frailty in oncogeriatrics, making it difficult to compare results across studies (21).

We also studied the prognostic value of the EFS, and we observed a significant association between the EFS and one-year mortality, both in univariate analysis and after adjustment for cancer type and the presence of an advanced stage. In Europe, despite advances in oncology medicine, cancer remains the second leading cause of mortality after cardiovascular diseases (36). A limited number of studies have explored the relationship between the EFS and mortality in older cancer patients (21, 25). Røyset et al. (21) recently showed that the EFS was an independent predictor of two-year mortality in older cancer patients receiving radiotherapy. In the same way, Meyers et al. (25) also observed that EFS score was inversely related to overall survival in 46 patients with colorectal receiving chemotherapy (5.2 months for EFS ≥7, compared to 15.4 months for EFS <7, *p* = 0.036).

The best predictive threshold value of the EFS for one-year mortality was evaluated as greater than or equal to 7/17 points, with a sensitivity of 72% and a specificity of 52%. A similar threshold was suggested by other authors although they evaluated mortality over more than 1 year (25). When using a screening test, clinicians aim to reach the highest possible sensitivity to avoid excess falsely negative frail patients, who would benefit from a more thorough evaluation, diagnostic testing, or treatment. The sensitivity for this threshold is close to the sensitivity found in the validation study of the G8 (16). However, since our study excluded patients with a normal G8 score, we likely excluded patients who had positive EFS and positive CGA and including them would have probably increased the sensitivity of the EFS. The specificity in our study was 52%. This means that the number of deaths must roughly be the same regardless of the Edmonton score, even though there is an association between the EFS and one-year mortality, persistent after adjusting for cancer type and stage.

As reported by several studies, cancer type and stage significantly influence mortality in oncology patients. Patients with pancreatic, liver, biliary, or lung cancer, as well as those with advanced disease, have higher mortality rates (1, 21, 37). Moreover, patients for whom the disease stage was unknown have higher mortality compared to those with localised disease. In a population-based prostate cancer registry, an unknown stage was increasing with age at diagnosis, and was associated with a poorer cancer-related survival than for those with a localised stage, but an improved survival compared to those with a metastatic stage, as in our study (38). This observation could have different explanations including the fact that some patients did not receive treatment despite an initial intention to treat them which justified their initial geriatric assessment, or they may not have undergone a full diagnostic workup due to their high level of frailty, or due to the patient’s refusal to be treated, or the staging was perhaps not necessary for the choice of treatment decision (39).

Our study has strengths and limitations. To our knowledge, there are very few studies comparing the performance of the EFS to that of the G8 (24, 40) This preliminary validation study suggests that the EFS may offer additional value in better identifying patients who would benefit from a CGA. Another strength of the study is the systematic inclusion of patients evaluated by a standardised geriatric assessment performed by the trained oncogeriatric teams working in two cancer referral hospitals. Finally, the total number of 380 patients included in our study exceeds the number included in the main studies focusing on the use of the EFS in oncogeriatrics, which ranged from 46 to 301 patients (20–26).

Our study also has several limitations. Due to its retrospective nature, some missing data limited a comprehensive evaluation of geriatric syndromes, such as IADL. Consequently, the total number of geriatric syndromes may have been underestimated. Similarly, we decided not to include the CIRS-G, given that cancer is included among the items scored. When data were missing for cognitive or mobility assessments, we considered the CGA domains as pathological if the record indicated that the test was not feasible, whether for cognitive reasons or functional reasons. It is possible that some patients were miscategorised due to the lack of information in the absence of an assessment. While our cohort is larger than those in many comparable studies, the diversity of cancers and treatments still led to limited representation of certain cancer subgroups.

This can be partly explained by the varying degrees of collaboration between the oncology specialists and the oncogeriatric team, rather than the epidemiological distribution of cancers in older people. For example, compared to the worldwide prevalence of cancers in 2022, there were more gastro-intestinal (34% vs. 25%) and urological (22% vs. 14%) cancers in our population, and similar rates of breast (12%) and lung (11%) cancers (36).

Finally, the heterogeneity of cancers and treatments is both a strength and a challenge of our study. It reflects the diversity of the population encountered in oncogeriatrics but also poses a significant challenge for establishing a uniform classification, especially in terms of advanced disease stages. Each type of solid tumour and hematological malignancy has its own staging system and specific prognostic indicators. We opted for a dichotomous classification into localised and advanced disease, in line with the ONCODAGE study definition, with the difference being that we included more diverse hematological malignancies, necessitating the use of specific prognostic scores for each type (16). In their study, Røyset et al. (21) chose to base their analyses on the oncologists’ treatment intentions (curative versus palliative), believing this approach might better represent the various prognostic factors of the cancers. This could have been an interesting alternative approach.

This preliminary study requires further validation through additional, prospective studies involving a larger sample of patients, including those with a non-pathological G8 score.

## 5 Conclusion

The Edmonton Frail Scale reliably improves the sensitivity to predict frailty as detected by the comprehensive geriatric assessment in older people with various types of cancer with different treatment objectives, that had been pre-selected as potentially frail by the G8 score. In addition to this, the EFS is quicker to administer and it has the advantage to evaluate geriatric domains not covered by the G8, such as cognitive function, physical performance, and social support. We observed a strong correlation between the EFS, and the number of pathological domains identified by the CGA, as well as between the EFS and the G8 score. The one-year mortality was influenced by the EFS score, even after adjusting for cancer type and disease stage, suggesting its predictive value for survival outcomes. This study suggests that the EFS could be a valuable tool for screening frailty in older cancer patients to help oncologists make the best decisions for the treatment of frail patients while avoiding unnecessary geriatric assessments. The use of the EFS could optimize healthcare resource utilization while ensuring targeted care for frail patients. Further studies are necessary to validate the EFS as a screening tool for older people with cancer, regardless of their G8 score.

## Data Availability

The raw data supporting the conclusions of this article will be made available by the authors, without undue reservation.
